# Exploring Psychosocial Mediators of the Associations of Lifetime Trauma and Body Mass Index in African American Women

**DOI:** 10.1089/heq.2020.0056

**Published:** 2020-12-30

**Authors:** Billy A. Caceres, Veronica Barcelona, Cindy Crusto, Jacquelyn Y. Taylor

**Affiliations:** ^1^ Center for Research on People of Color, Columbia University School of Nursing, New York, NY, USA.; ^2^ Yale University School of Nursing, Orange, CT, USA.; ^3^ Yale University School of Medicine, New Haven, CT, USA.

**Keywords:** African American, women, BMI, trauma

## Abstract

**Purpose:** This study sought to examine the association between lifetime trauma (i.e., childhood, adulthood, and cumulative) and body mass index (BMI) and if this association was mediated by psychosocial factors (i.e., depressive symptoms and stress) in a sample of African American women.

**Methods:** We examined lifetime trauma among African American women in the Intergenerational Impact of Genetic and Psychological Factors on Blood Pressure Study (InterGEN) study. We conducted mediation analyses with bootstrapping to assess the direct and indirect effects of increasing forms of trauma across the lifespan on BMI. Depressive symptoms and stress were examined as mediators of these associations.

**Results:** The analytic sample included 138 women with a mean age of 31.9 years. Approximately half of women reported any childhood trauma (47.8%) and more than half (73.2%) reported any adulthood trauma. The direct effects of all forms of trauma were associated with greater depressive symptoms. Only lifetime trauma was associated with higher stress overload (*B*=2.40, standard error [SE]=1.12, *p*<0.05). Higher depressive symptoms were associated with higher BMI in all models. The indirect effects of adulthood trauma (*B*=0.60, SE=0.20, *p*<0.01) and lifetime trauma (*B*=0.53, SE=0.20, *p*<0.01) on BMI were partially mediated by depressive symptoms.

**Conclusion:** These findings indicate that depressive symptoms mediate the associations between adulthood and lifetime trauma with BMI. Interventions aimed at reducing elevated BMI in African American women should account for the influence of depressive symptoms. Future research should replicate these analyses in other samples of African American women.

## Introduction

Obesity, defined as body mass index (BMI) ≥30 kg/m^2^, is a prevalent and important indicator of health and risk of future disease. Obesity is a common comorbidity for people with hypertension, diabetes, and metabolic syndrome, and these often result in increased cardiovascular disease risk.^1^ In 2018, the age-adjusted prevalence of obesity among U.S. adults was 42.4% and rates were highest among African American women (56.9%) when stratified by race and sex.^2^ The etiology of obesity is complex and multifactorial, including genetic, environmental, and cultural influences.^3,4^ African American women may be particularly at risk for obesity owing to factors such as cultural acceptance of higher body weight, residential segregation, health literacy, and lower socioeconomic status.^5^

There is growing evidence that traumatic experiences across the lifespan are associated with elevated BMI in adults. A meta-analysis of 41 studies found that adverse childhood experiences (ACEs) were associated with increased risk of elevated BMI across the lifespan and this association was stronger in samples that included more women.^6^ Similarly, a systematic review of 36 studies reported a significant association between childhood abuse and adult obesity.^7^ Analyses of data from the Black Women's Health Study found that childhood abuse was associated with increased risk for elevated overall and central obesity in African American women.^8^ Another population-based study found that racial and ethnic minority women (e.g., African American, Latina) with a history of childhood physical abuse were more than twice as likely to meet criteria for obesity than those who reported no childhood physical abuse.^9^

Although most of the research on the associations of trauma and BMI has focused on experiences in childhood, there is evidence that trauma in adulthood is also associated with elevated BMI among women. For instance, a study conducted in Norway found that intimate partner violence (IPV) was associated with greater abdominal obesity, but no association with BMI was observed.^10^ In addition, analyses of data from the 2005 Behavioral Risk Factor Surveillance System in Missouri found that women with a history of IPV had higher rates of obesity than women with no history of IPV.^11^

Previous studies have identified psychosocial factors as potential mediators of the associations between trauma and BMI. A systematic review found that depressive symptoms were significant mediators of the associations of ACEs and elevated BMI in the majority of included studies.^12^ However, sex differences were not described. Among women, analyses of longitudinal data found that those who experienced adult IPV and reported depressive mood had accelerated 5-year weight gain compared with women who did not have depressive mood.^13^ Similarly, analyses of data from the National Survey of American Life found that African American women with a history of severe physical adult IPV were more than twice as likely to report having a depressive or anxiety disorder in their lifetimes and more than three times as likely to have post-traumatic stress disorder.^14^

Previous work highlights the importance of examining risk and protective factors that account for health disparities *within* vulnerable populations.^15^ Research that examines factors associated with elevated BMI among African American women can better elucidate the etiology of obesity in this population and inform the development of behavioral interventions aimed at African American women who are most at risk for elevated BMI. Despite a higher prevalence of traumatic experiences in childhood (i.e., parental IPV, household incarceration history, and household drug abuse^16^) and adulthood^17–19^ and higher rates of elevated BMI,^2^ to date, there has been limited investigation of the association of trauma and BMI in African American women.

Therefore, the purpose of this study was to examine the association between lifetime trauma and BMI, and if this association was mediated by psychosocial factors (i.e., depressive symptoms and stress) in a sample of African American women enrolled in the Intergenerational Impact of Genetic and Psychological Factors on Blood Pressure Study (InterGEN) study. Based on existing evidence, we hypothesized that trauma across the lifespan would be associated with higher BMI and that psychosocial factors would mediate this association.

## Materials and Methods

### Sample

The InterGEN study was a longitudinal cohort study conducted in Connecticut, examining the gene-environment effects of parenting stress, experiences of racial discrimination, and maternal depression on blood pressure in a cohort of African American women and children. Recruitment took place from April 2015 to September 2018. Mother/child dyads were recruited from early care and education programs, primary care clinics, and community events (i.e., health fairs). Eligibility criteria for enrolled mothers (*n*=250) included: (1) age ≥21 years; (2) self-identified as African American or Black; (3) spoke English; (4) did not report a psychiatric or cognitive disorders that would interfere with their ability to self-report experiences; and (5) enrolled with a biological child aged 3–5 years. Full study procedures for the InterGEN study have been described elsewhere.^20,21^

Trained research assistants verified eligibility and obtained written, informed consent from participants. InterGEN consisted of four study visits over ∼2 years, each 6 months apart. Clinical measurements of blood pressure, height, and weight were collected from both mother and child (Time 1 to Time 4). For the purposes of this study, we examined lifetime trauma exposure and covariates of demographics variables, alcohol and tobacco use, and coping strategies collected at Time 1 only. Mediators, such as symptoms of depression and stress overload, were measured at Time 2, whereas BMI was taken at Time 3. Demographic information and additional health and psychological measures were collected using Audio Computer-Assisted Self-Interview (ACASI) software. Study procedures were approved by Yale University's Institutional Review Board.

### Measures

#### Lifetime trauma

We used the Life Events Checklist (LEC) to assess exposure to trauma across the lifespan at baseline (Time 1).^22^ The LEC is a 17-item instrument to screen for traumatic events in each participants' lifetime. The LEC assesses exposure to 16 events known to result in post-traumatic stress and one additional item that assesses any other extraordinarily stressful event not captured in the first 16. For each traumatic event endorsed by a participant, they were asked how old they were the first time and the last time they experienced this event. We used responses to those two items to create a count of *childhood trauma* (range 0–7) representing events that occurred before the age of 18 years and *adulthood trauma* (range 0–8) representing events that occurred at age 18 years or older. We then summed *childhood trauma* and *adulthood trauma* to determine cumulative *lifetime trauma* (range 0–9) reported by participants.

#### Covariates

Covariates included demographic characteristics, health behaviors, and coping strategies; all measured at Time 1. The demographic characteristics we assessed were age, Latina ethnicity, education, and annual household income. We assessed tobacco and alcohol use in the past 3 months. In addition, we used the Coping Strategy Indicator (CSI) administered at Time 1 to assess self-report of strategies in response to a stressful event in the past 6 months.^23^ The CSI includes three subscales to assess different forms of coping in the past 6 months. Cronbach's alpha for each subscale of the CSI in this study were as follows: avoidance=0.80, seeking social support=0.92, and problem solving=0.93.

#### Mediators

We assessed depressive symptoms and stress overload as potential mediators of the association between lifetime trauma and BMI; both measured at Time 2. The Beck Depression Inventory (BDI) was used to assess depressive symptoms (e.g., sadness, agitation, and sleep loss).^24^ The BDI includes 21 items scored on severity of each symptom (0=low; 3=high). A total depression score is calculated by summing all responses (range 0–63) with higher scores indicating greater depressive symptoms. Cronbach's alpha in the present sample was 0.88. The Stress Overload Scale (SOS) was used to measure stress. Participants are asked to rate their stress on 24 items related to personal vulnerability (e.g., feelings of powerlessness, inadequacy, and debility) and event load (e.g., burden of outside demands, responsibilities, and pressures) in the past week.^25^ Each item is scored of a five-point Likert scale from not at all (1) to a lot (5). An overall score is calculated by adding scores of the personal vulnerability and event load subscales (range=24–120) with higher scores indicating greater stress. Cronbach's alpha in the present sample was 0.96.

### Body mass index

BMI (kg/m^2^) at Time 3 was calculated from height measured by portable stadiometer (Seca Corp., Hanover, MD), and weight by electronic scale (Tanita, Tokyo, Japan).

### Statistical analyses

Analyses were conducted in Mplus Version 7. We first excluded participants with missing data for lifetime trauma at Time 1 (independent variable) and BMI at Time 3 (dependent variable). We used independent sample's *t*-test and chi-square tests to assess differences in study variables by report of any lifetime trauma versus no lifetime trauma. A significance level of 0.05 was selected *a priori*. Next, we used path analysis to separately assess the direct and indirect effects of increasing forms of trauma across the lifespan (i.e., childhood, adulthood, and lifetime) on BMI adjusted for age, ethnicity, education, annual household income, alcohol and tobacco use, and the three coping strategy subscales (i.e., avoidance, seeking social support, and problem solving). Bootstrapping was used to obtain robust standard errors and confidence intervals for indirect effects. This permits significance testing of the indirect effects of trauma on BMI.^26^ We used 1000 bootstraps to generate confidence intervals of the indirect effects of trauma across the lifespan on BMI through psychosocial factors (i.e., depressive symptoms and stress). This provided results for the direct, indirect, and total effects of increasing forms of trauma with BMI. Model fit was assessed with the root mean square error of approximation (RMSEA) and confirmatory factor index (CFI). Models with an RMSEA <0.05 are preferred.^27^ The CFI is widely used to assess model fit with a value >0.95 indicating good fit.^28^ Missing data were handled using full information maximum likelihood, which is the default method for handling missing data in Mplus.

## Results

We excluded women with missing data for lifetime trauma (*n*=9) at Time 1 and BMI at Time 3 (*n*=88). We also excluded women who responded “don't know” or “refused” to covariates included in this study (*n*=15). The final sample comprised 138 women. Results of analyses examining differences in study variables by lifetime trauma are presented in Table 1. The mean age of women in the sample was 31.9 years, 14 (10.1%) were Latina, 25 (18.1%) had completed a Bachelor's degree or greater, and 27 (19.6%) had a household income less than $5000. Overall, 66 (47.8%) women reported any childhood trauma, 101 (73.2%) women reported any trauma in adulthood, and 102 (73.9%) women reported any lifetime trauma. There were few statistically significant differences across study variables by lifetime trauma. However, women who reported any lifetime trauma had higher depressive symptom scores (6.0 vs. 3.5, *p*<0.05) and higher scores on the problem-solving coping subscale (27.8 vs. 24.8, *p*<0.05) compared with women with no lifetime trauma. There was no significant difference in BMI between women with and without a history of lifetime trauma.

**Table 1.** Sample Characteristics of African American Women With and Without a History of Lifetime Trauma (*N*=138)

	No lifetime trauma (n*=36), *n (%)	Any lifetime trauma (n*=102), *n (%)	Total sample (N*=138), *n (%)
Demographic characteristic at Time 1			
Mean, age (SD)	32.2 (5.6)	31.9 (5.9)	31.9 (5.8)
Latina ethnicity			
Yes	2 (5.6)	12 (11.8)	14 (10.1)
No	34 (94.4)	90 (88.2)	124 (89.9)
Education			
≤High school or GED	15 (41.7)	37 (36.3)	52 (37.7)
Some college or Associate's degree	17 (47.2)	44 (43.1)	61 (44.2)
≥ Bachelor's degree	4 (11.1)	21 (20.6)	25 (18.1)
Annual household income			
<$5000	8 (22.2)	19 (18.6)	27 (19.6)
$5000–$24,999	17 (47.2)	44 (43.1)	61 (44.2)
$25,000–$49,999	7 (19.4)	26 (25.5)	33 (23.9)
$50,000–$74,999	3 (8.3)	6 (5.9)	9 (6.5)
≥$75,000	1 (2.8)	7 (6.9)	8 (5.8)
Tobacco use in past 3 months			
Yes	7 (19.4)	20 (19.6)	27 (19.6)
No	29 (80.6)	82 (80.4)	111 (80.4)
Alcohol use in past 3 months			
Never	17 (47.2)	45 (44.1)	62 (44.9)
Once or twice	13 (36.1)	29 (28.4)	42 (30.4)
Monthly	5 (13.9)	17 (16.8)	22 (15.9)
Weekly	1 (2.8)	10 (9.8)	11 (8.0)
Daily or almost daily	0 (0.0)	1 (0.9)	1 (0.7)
Mean coping strategy indicator (SD)			
Avoidance (range 11–31)	18.2 (4.9)	19.8 (4.6)	19.4 (4.7)
Seeking social support (range 11–33)	21.3 (6.7)	23.5 (6.2)	22.9 (6.4)
Problem solving (range 15–33)	24.8 (6.6)	27.8 (5.4)^*^	27.0 (5.8)
Lifetime trauma at Time 1			
Any lifetime trauma	—	—	102 (73.9)
Any childhood trauma	—	—	66 (47.8)
Any adulthood trauma	—	—	101 (73.2)
Psychosocial mediators at Time 2			
Mean Beck Depression Inventory (SD)	3.5 (4.1)	6.0 (6.5)^*^	5.4 (6.2)
Mean Stress Overload Scale (SD)	46.7 (24.4)	56.1 (24.1)	54.0 (24.4)
BMI at Time 3			
BMI (kg/m^2^)	30.8 (8.7)	30.6 (8.4)	30.7 (8.5)
Weight status			
Underweight	0 (0.0)	6 (5.9)	6 (4.3)
Normal	6 (16.7)	19 (18.6)	25 (18.1)
Overweight	14 (38.9)	29 (28.4)	43 (31.2)
Obese	16 (44.4)	48 (47.1)	64 (46.4)

Sample sizes may vary owing to missing data, ^*^p<0.05.

BMI, body mass index; SD, standard deviation.

Results of mediation analyses examining the direct and indirect effects of reporting increasing forms of trauma on BMI in African American women are given in Table 2. All models had adequate fit. The direct effects of all forms of trauma were associated with greater depressive symptoms (childhood trauma *B*=1.10, standard error [SE]=0.54, *p*<0.05); adulthood trauma (*B*=1.01, SE=0.30, *p*<0.01); and lifetime trauma (*B*=0.90, SE=0.26, *p*<0.001). Only lifetime trauma was associated with higher stress overload (*B*=2.40, SE=1.12, *p*<0.05). Higher depressive symptoms were associated with significantly higher BMI in all models (childhood trauma *B*=0.58, SE=0.18, *p*<0.001; adulthood trauma *B*=0.60, SE=0.17, *p*<0.01; and lifetime trauma *B*=0.58, SE=0.18, *p*<0.01). In contrast, higher stress overload was associated with lower BMI for all forms of trauma. The associations between the direct effects of trauma across the lifespan on BMI were not statistically significant. However, the indirect effects of adulthood trauma (*B*=0.60, SE=0.20, *p*<0.01) and lifetime trauma (*B*=0.53, SE=0.20, *p*<0.01) on BMI were partially mediated by depressive symptoms. Stress overload did not mediate the associations of different forms of trauma across the lifespan with BMI. Finally, the total effect of trauma across the lifespan on BMI was not statistically significant.

**Table 2.** Direct and Indirect Effects of Increasing Forms of Trauma Across the Lifespan on Body Mass Index in African American Women (*N*=138)

	Childhood trauma (range 0–7)	Adulthood trauma (range 0–8)	Lifetime trauma (range 0–9)
**Paths**	**Beta (SE)**		
Direct effects			
Trauma → depressive symptoms	1.10 (0.54)^*^	1.01 (0.30)^**^	0.90 (0.26)^***^
Trauma → stress overload	1.97 (1.53)	2.67 (1.37)	2.40 (1.12)^*^
Depressive → BMI	0.58 (0.18)^***^	0.60 (0.17)^**^	0.58 (0.18)^**^
Stress overload → BMI	−0.08 (0.04)^*^	−0.08 (0.04)^*^	−0.08 (0.04)^*^
Trauma → BMI	−0.35 (0.62)	−0.40 (0.39)	−0.26 (0.34)
Indirect effects			
Trauma → depressive symptoms → BMI	0.64 (0.36)	0.60 (0.20)^**^	0.53 (0.20)^**^
Trauma → stress overload → BMI	−0.16 (0.15)	−0.21 (0.15)	−0.19 (0.13)
Total indirect effect	0.48 (0.32)	0.39 (0.19)^*^	0.33 (0.17)^*^
Total effect	0.13 (0.64)	−0.02 (0.36)	0.08 (0.33)
Trauma → BMI			

All models adjusted for age, ethnicity, education, household income, tobacco use, alcohol use, and coping strategies.

^*^ *p*<0.05; ^**^p<0.01; ^***^p<0.001.

SE, standard error.

## Discussion

To our knowledge, this is one of the few studies to assess psychosocial mediators of the association between trauma and BMI in a community-based sample of African American women. Given limited evidence of the influence of trauma on BMI in African American women, this work provides an important contribution to this area of study. Our results contradict previous analyses of population-based data that found childhood trauma had a direct effect on elevated BMI in African American women.^8,9^ It is important to note that although previous studies focused on interpersonal violence (i.e., physical and sexual abuse) in childhood, we examined several forms of trauma across the lifespan (e.g., natural disasters, military combat, transportation accidents, and crimes). This may explain the differences between our findings and previous work. However, when we conducted exploratory analyses examining the associations of physical and sexual abuse in childhood and adulthood with BMI we found no direct effects of interpersonal violence on BMI. Future studies with African American women should examine whether the associations of trauma and BMI differ between interpersonal violence and other forms of trauma and whether these associations differ between population-based and community-based samples.

Overall, we found that increases in the number of traumatic events in childhood or adulthood had a negative influence on mental health in this vulnerable population. Although trauma did not have a direct or total effect on BMI in African American women, we identified that depressive symptoms partially mediated the associations of adulthood and lifetime trauma with BMI. Consistent with analyses of nationally representative data, we found that traumatic events across the lifespan were associated with worse mental health in African American women.^8,14^ For instance, analyses of longitudinal data from the Black Women's Health Study found that severe physical or sexual abuse in childhood was positively associated with depressive symptoms in adulthood.^8^ Similarly, analyses of cross-sectional data from the National Survey of American Life (*N*=505) found that African American women with a history of physical IPV were more than twice as likely to have depression in their lifetimes.^14^ In our study, depressive symptoms emerged as a mediator of the associations between adulthood and lifetime trauma with BMI.

Contrary to our hypotheses, stress was not a significant mediator of the associations of different forms of trauma and BMI in African American women. Only lifetime trauma was associated with higher stress overload and stress overload had a negative direct effect on BMI. Perhaps a measure of stress directly related to trauma, such as post-traumatic stress, would have been a more appropriate mediator to examine. Indeed, previous work has identified post-traumatic stress as a risk factor for elevated BMI in women^29–31^; however, this measure was not assessed in the InterGEN study. Additional psychosocial risk factors (e.g., post-traumatic stress and anxiety) should be examined as potential mediators of the association of trauma and BMI in African American women in future studies.

These results underscore the need for future research examining the association of trauma across the lifespan with BMI and other cardiometabolic risk factors in African American women. It is recognized that conducting research *within* vulnerable groups is important to identify those individuals most at risk for adverse health outcomes.^15^ Therefore, research that replicates the analyses conducted in this study is needed to determine if findings are consistent across different samples of African American women. Future research in this area will help identify subgroups of African American women most at risk for adverse health outcomes related to elevated BMI. Alternatively, there is a need to examine whether the associations described in our study differ across racial and ethnic groups. Comparisons between African American women and women of other races/ethnicities may elucidate potential differences in the associations of trauma and BMI. Overall, there is a lack of evidence-based trauma-informed strategies for the prevention of obesity in women who have experienced trauma. In fact, a recent systematic review found no interventions for the treatment of elevated BMI in women with a history of childhood trauma.^12^ This suggests a significant knowledge gap that warrants further investigation. Given this evidence, our findings can inform future work that targets specific psychosocial risk factors for elevated BMI in African American women.

This study has several implications for clinical practice. Although recent guidance from the U.S. Preventative Services Task Force only recommends screening for IPV and other forms of trauma among women of reproductive age,^32^ the World Health Organization^33^ and the American College of Obstetricians and Gynecologists recommend routine screening for IPV among all women.^34^ In clinical settings, it is important for clinicians to provide women who have experienced traumatic events with proper mental health treatment. In particular, we identify a need to target depressive symptoms in African American women who have experienced traumatic events, which may help mitigate its negative effects on BMI. Clinicians should also be educated about the negative health effects of trauma in African American women and relevant psychosocial factors to understand this association.

### Strengths and limitations

This study has both strengths and weaknesses. A major strength of this study is that it is a cohort study of all African American women, which allows us to examine questions unique to this population. This study adds to the limited literature on the health effects of trauma in African American women. One limitation is that we used the LEC rather than instruments designed to assess traumatic events at specific life stages (e.g., ACEs scale). Also, additional mediators of the associations of trauma and BMI were not included. Next, despite a limited sample size, we observed differences in mediators under study. However, future studies with larger sample sizes that allow for mediation analyses with a greater number of psychosocial mediators are needed. Given that the InterGEN study focused on mother/child dyads, our findings may not be generalizable to African American women who are not mothers. Future work is needed that include larger samples of African American and racially diverse women to increase the reliability and generalizability of findings. Finally, future studies should examine the epigenetic effects of trauma on BMI in women with African ancestry.

## Conclusions

In summary, we observed statistically significant associations between trauma with depressive symptoms and stress (through direct effects) and between trauma and BMI (through indirect effects) among African American women. Depressive symptoms partially mediated the associations between adulthood and lifetime trauma with BMI. These findings have important implications for providing trauma-informed care to African American women, and may inform future areas of inquiry for this understudied population.

## Abbreviations Used

ACE: adverse childhood experience
BDI: Beck Depression Inventory
BMI: body mass index
CFI: confirmatory factor index
CSI: Coping Strategy Indicator
InterGEN: Intergenerational Impact of Genetic and Psychological Factors on Blood Pressure Study
IPV: intimate partner violence
LEC: life events checklist
RMSEA: root mean square error of approximation
SD: standard deviation
SE: standard error
